# Tumour-specific arginine vasopressin promoter activation in small-cell lung cancer

**DOI:** 10.1038/sj.bjc.6690623

**Published:** 1999-08

**Authors:** J M Coulson, J Stanley, P J Woll

**Affiliations:** CRC Department of Clinical Oncology, University of Nottingham, City Hospital, Hucknall Rd, Nottingham, NG5 1PB, UK

**Keywords:** vasopressin, promoter, SCLC, transcription, tumour-specific, enhancer

## Abstract

Small-cell lung cancer (SCLC) can produce numerous mitogenic neuropeptides, which are not found in normal respiratory epithelium. Arginine vasopressin is detected in up to two-thirds of SCLC tumours whereas normal physiological expression is essentially restricted to the hypothalamus. This presents the opportunity to identify elements of the gene promoter which could be exploited for SCLC-specific targeting. A series of human vasopressin 5′ promoter fragments (1048 bp, 468 bp and 199 bp) were isolated and cloned upstream of a reporter gene. These were transfected into a panel of ten cell lines, including SCLC with high or low endogenous vasopressin transcription, non-SCLC and bronchial epithelium. All these fragments directed reporter gene expression in the five SCLC cell lines, but had negligible activity in the control lines. The level of reporter gene expression reflected the level of endogenous vasopressin production, with up to 4.9-fold (s.d. 0.34) higher activity than an SV40 promoter. The elements required for this strong, restricted, SCLC-specific promoter activity are contained within the 199-bp fragment. Further analysis of this region indicated involvement of E-box transcription factor binding sites, although tumour-specificity was retained by a 65-bp minimal promoter fragment. These data show that a short region of the vasopressin promoter will drive strong expression in SCLC in vitro and raise the possibility of targeting gene therapy to these tumours. © 1999 Cancer Research Campaign


*British Journal of Cancer* (1999) **80**(12), 1935–1944
© 1999 Cancer Research Campaign
Article no. bjoc.1999.0623

### Tumour-specific arginine vasopressin promoter activation in small-cell lung cancer

**JM Coulson, J Stanley and PJ Woll**

CRC Department of Clinical Oncology, University of Nottingham, City Hospital, Hucknall Rd, Nottingham NG5 1PB, UK

**Summary** Small-cell lung cancer (SCLC) can produce numerous mitogenic neuropeptides, which are not found in normal respiratory epithelium. Arginine vasopressin is detected in up to two-thirds of SCLC tumours whereas normal physiological expression is essentially restricted to the hypothalamus. This presents the opportunity to identify elements of the gene promoter which could be exploited for SCLC-specific targeting. A series of human vasopressin 5′ promoter fragments (1048 bp, 468 bp and 199 bp) were isolated and cloned upstream of a reporter gene. These were transfected into a panel of ten cell lines, including SCLC with high or low endogenous vasopressin transcription, non-SCLC and bronchial epithelium. All these fragments directed reporter gene expression in the five SCLC cell lines, but had negligible activity in the control lines. The level of reporter gene expression reflected the level of endogenous vasopressin production, with up to 4.9-fold (s.d. 0.34) higher activity than an SV40 promoter. The elements required for this strong, restricted, SCLC-specific promoter activity are contained within the 199-bp fragment. Further analysis of this region indicated involvement of E-box transcription factor binding sites, although tumour-specificity was retained by a 65-bp minimal promoter fragment. These data show that a short region of the vasopressin promoter will drive strong expression in SCLC in vitro and raise the possibility of targeting gene therapy to these tumours.

**Keywords:** vasopressin; promoter; SCLC; transcription; tumour-specific; enhancer

Lung cancer is the most common fatal malignancy in the developed world and small-cell lung cancer (SCLC) comprises about 25% of these tumours. SCLC characteristically secrete a variety of neuropeptides, such as adrenocorticotrophin hormone (ACTH), gastrin-releasing peptide (GRP), gastrin, cholecystokinin and arginine vasopressin (AVP). Many of these peptides can act as autocrine growth factors (Woll, 1996). AVP is the peptide most commonly secreted by SCLC, with up to 67% of tumours showing immunoreactivity (Friedmann et al, 1994) and elevated plasma concentrations (Maurer et al, 1983). In contrast, AVP production is rarely detected in non-neuroendocrine lung carcinomas (Friedmann et al, 1993). Vasopressin receptors have been demonstrated in SCLC (Fay et al, 1994; Coulson et al, 1997; North et al, 1998) and AVP is mitogenic for SCLC (Sethi and Rozengurt, 1991) suggesting that it can act as an autocrine factor promoting proliferation in these tumours.

Physiological AVP expression is largely restricted to the hypothalamus and is tightly regulated in response to plasma osmolality. In contrast, the ectopic secretion of AVP by SCLC can be associated with the clinical syndrome of dilutional hyponatraemia (Johnson et al, 1997). Because expression of AVP is so restricted in normal tissue, and expression of AVP in SCLC does not respond to osmolality, transcription from the AVP gene promoter in SCLC may be uniquely tumour-specific. In other tumour types, tissue-specific promoters have been used to drive expression of a therapeutic gene (Huber, 1991; Vile and Hart, 1993), therefore we propose that the AVP promoter could be exploited for targeting gene therapy to SCLC.

Approximately 1.2-kb of the human AVP promoter region has been sequenced (Bahnsen et al, 1992). The 200-bp sequence immediately 5′ to the coding region shows high cross-species homology (Sausville et al, 1985; Hara et al, 1990) and some regulatory elements within this proximal promoter region have been identified, for example those mediating cAMP responsiveness (Verbeeck et al, 1991; Pardy et al, 1992). However, no elements have yet been described which confer tissue-specificity on AVP expression. Transgenic studies indicate that large regions of both the 5′ and 3′ untranslated regions are required to mimic normal hypothalamic AVP expression (Ang et al, 1993). The functional human promoter might therefore extend further upstream than the partially characterized proximal region, or contain alternative SCLC-specific elements. We have isolated regions of the human AVP promoter, and an enhancer, for evaluation of reporter gene expression to determine the promoter elements relevant for tumour expression. We have identified a short fragment of the AVP promoter with strong activity in vitro and selectivity for SCLC cell lines, which could be developed for targeting therapy to this tumour type.

### MATERIALS AND METHODS

### Computer analysis

Sequence analysis software was accessed via the UK HGMP-RC, Cambridge. Transcription factor binding sites within the AVP promoter and gene were predicted using ‘Promoter Scan’ (Prestridge, 1995) and ‘Signal Scan’ (Prestridge, 1996).

*Received 2 October 1998*
*Revised 16 February 1999*
*Accepted 17 February 1999*

*Correspondence to:* JM Coulson

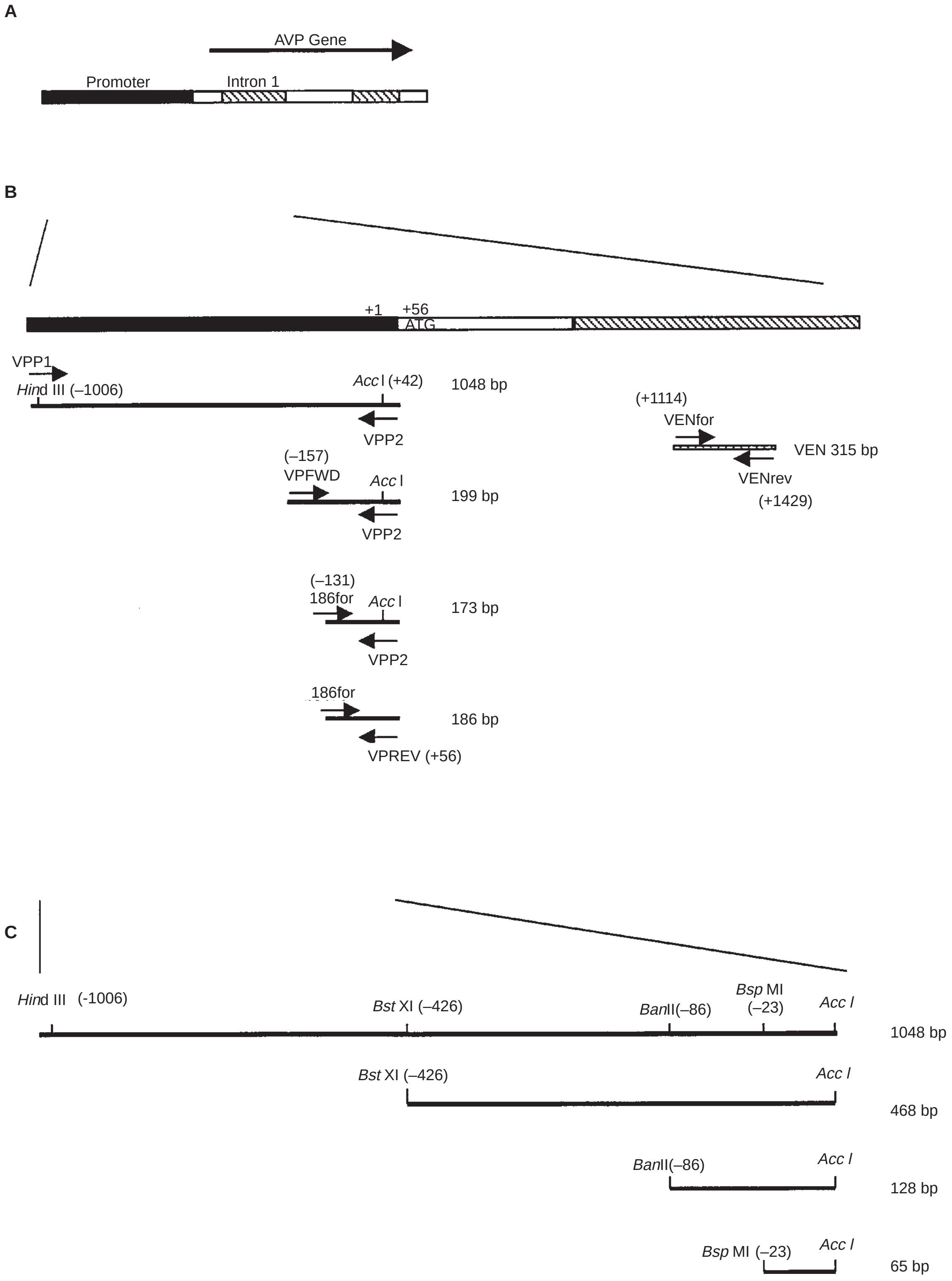

**Figure 1** PCR amplification and cloning of human AVP promoter and enhancer elements. (**A**) Map of the AVP structural gene comprising the promoter, three exons and two introns. (**B**) AVP promoter and enhancer fragments cloned from this region by PCR. The promoter, exon 1 and intron 1 are shown enlarged (top) with the translational start codon at +56 relative to the transcriptional start site (+1). The PCR primer pairs and the promoter regions amplified are shown below, the sizes given are for the cloned products. The VEN enhancer was amplified from the first intron by VENfor / VENrev (+1114 to +1429). (**C**) Additional constructs (3′ end at +42) were generated using restriction sites within the 1048-bp region, which is shown expanded (top). The *Bst*X1 (–426), *Ban*II (–86) and *Bsp*M1(–23) sites represent the 5′ ends of clones p468, p128 and p65 respectively

*© 1999 Cancer Research Campaign*

### Isolation of AVP promoter and enhancer regions

The AVP gene 5′ region and a potential enhancer within the first intron were amplified from NCI-H510 (SCLC) genomic DNA. All PCR primers were from Cruachem (Glasgow, UK) or MWG-Biotech (Germany). Dynazyme II polymerase (Flowgen, Staffs, UK) was used in optimized buffer (Flowgen) with 30 standard PCR cycles and a final 10-min extension at 72°C to facilitate T/A cloning. The primers VPFWD (5′ CAG ACA GGC CCA CGT GTG 3′) and VPREV (5′ CCT GGT GCA CAC AGG TGG ACC 3′) amplified the proximal 199-bp fragment; VPP1 (5′CCT CTT TAG ACC TGC *AAG CTT* GGG 3′) and VPP2 (5′ CCT GGT GCA CAC *AGG TCG ACC* C 3′) amplified a large fragment, of 1048 bp when cloned using the *Hin*dIII and *Acc*I restriction sites engineered into the primer sequences (underlined above). The primer VP186for (5′ TG CCT GAA TCA CTG CTG ACG GC 3′) was used with VPREV to amplify a 186 bp PCR product, and with VPP2 to amplify a 186 bp product containing an *Acc*I site. The enhancer was obtained using VENfor (5′ CTC TCC GCA TGG TGT AGT GG 3′) and VENrev (5′ TGC GGC TGC AGG CAC GCT CG 3′). The primer locations and products are shown in Figure 1B.

### Construction of reporter plasmids

The PCR products described above were ligated into a T/A cloning vector derived from an *Eco*RV digest of pBluescript (Stratagene), and their identity and sequence confirmed by automated sequencing. No mutations relative to the previously published sequences (Sausville et al, 1985; Bahnsen et al, 1992) were identified. Restriction sites, in the primers or plasmid, were utilized for sub-cloning into chloramphenicol acetyl transferase (CAT) reporter constructs. The promoterless (pnone) plasmid pBLCAT6 (a gift from Prof. Ian Hart, ICRF/Richard Dimbleby Labs, St Thomas' Hospital, London, UK) was used to clone the 5′ fragments generating constructs with different lengths of promoter: p1048, p199, p186 and p173. The plasmid p186MUT was derived by cloning the 173 PCR product without using the internal *Acc*I site in the VPP2 primer. Additional constructs, p468, p128 and p65 (Figure 1C), were generated from p1048 by restriction digestion with *Bst*X1/*Hin*dIII, *Ban*II/*Hin*dIII and *Bsp*M1/*Hin*dIII respectively, then blunt-ended with T4 DNA polymerase (New England Biolabs, Herts, UK) and ligated. The enhancer element was cloned in the forward orientation into pBLCAT6 (promoterless) and pBLCAT5 (containing a minimal thymidine kinase (tk) promoter) to produce pVEN and pVENtk respectively. Plasmid pVEN468 was generated by sub-cloning of the VEN PCR product into p468 using the *Bst*X1/*Hin*dIII sites, placing the enhancer upstream of the intermediate length AVP promoter.

### Cell lines and culture

The Lu-165 SCLC line was a gift from Dr T Terasaki (National Cancer Centre, Tokyo, Japan), the non-SCLC (NSCLC) lines MOR/P and COR-L23 were a gift from Dr P Twentyman (established at the MRC Clinical Oncology Research Unit, Cambridge, UK) and the SCLC line NCI-H711 was a gift from Dr B Johnson (NCI, Bethesda, MD, USA). Other SCLC and NSCLC cell lines originated from the ATCC. These cell lines were routinely maintained in RPMI–10% bovine calf serum (BCS) at 5% carbon dioxide 37°C. Primary normal human bronchial epithelial cells (NHBE) from a single donor (4653, Clonetics) and SV40 transformed HBE (a gift from Dr W Franklin, University of Colorado Cancer Centre, USA) were maintained in fully supplemented Bronchial Epithelial Growth Medium (Clonetics) and used at the third passage. All cells were used during exponential growth for experiments.

### Isolation of AVP mRNA from cultured cells and tissues

Total cellular RNA was prepared from $2 \times 10^7$ cultured cells using a Purescript RNA kit (Gentra Systems Inc.) and prepared from human hypothalamus using TRIzol (Life Technologies Inc.). The concentration and integrity of all total RNA was determined. Poly($A^+$) mRNA was isolated from $2 \times 10^6$ cultured cells using Dynabeads mRNA Direct (Dynal) and resuspended in a volume of 50 μl.

### RT-PCR

For reverse transcription polymerase chain reaction (RT-PCR), 1 μg of total cellular RNA was reverse transcribed (Promega, UK). The cDNA was denatured and diluted to 100 μl, 2 μl of this was used for PCR. Dynazyme II polymerase was used in magnesium-free buffer (Flowgen) supplemented to a final concentration of 1 mM magnesium chloride. Glyceraldehyde-3-phosphate dehydrogenase (GAPDH) primers (GAPDHfor: 5′ CCA ACC ATG GCA AAT TCC ATG GCA 3′/GAPDHrev: 5′ TCT AGA CGG CAG GTC AGG TCC ACC 3′, 28 cycles), which produce a band of 598 bp, were used as a control (Maier et al, 1990). AVP expression was detected by standard or nested PCR. In the nested PCR, the first round (25 cycles) with primers VPRNAfor (5′ TGC ATA CGG GGT CCA CCT GT 3′) and VPex2rev (5′ GTT GCA GCA AAC GCC GAA GG 3′) would generate a 348 bp product. A 3 μl aliquot of this was then reamplified with two internal primers VPRNAfor2 (5′ CAT GCT GCC CGC CTG CTT CC 3′) and VASrev (5′ TAG TTC TCC TCC TGG CAG C 3′) which generated a 227 bp product spanning intron 1. For the standard PCR protocol, the primers VPRNAfor and VASrev generated a 269 bp product (35 cycles).

### Northern blotting

Northern blots were prepared from glyoxal treated RNA. Approximately 40 μg of total RNA or the 50 μl preparation of poly($A^+$) RNA were loaded per lane. Gels were electrophoresed in 10 mM sodium phosphate (pH 7.0) with recirculation of the buffer. RNA was capillary transferred onto Hybond-N (Amersham), fixed by ultraviolet (UV) irradiation and baked at 80°C. The RNA markers (Sigma) were stained with methylene blue. Random primed probes were generated from double-stranded DNA, corresponding to exon 1 of the AVP gene, and a cloned fragment of GAPDH, using [$\alpha^{32}$P]dCTP and an oligolabelling kit (Pharmacia). Blots were prehybridized for 2 h in 6 × sodium–saline citrate (SSC), 1 × Denhardts' (0.2% Ficoll 400, 0.2% polyvinylpropylene, 0.2% BSA), 10 mM sodium phosphate, 1 mM EDTA pH 8.0, 5% dextran sulphate and 25 μg $ml^{-1}$ denatured salmon sperm DNA; then hybridized with the probe at 65°C for 16 h. Blots were washed to 2 × SSC and exposed to autoradiography film for 1 day (GAPDH) and 6 or 55 days (AVP, for total and poly(A) blots respectively).

*© 1999 Cancer Research Campaign*

### Immunocytochemistry

Intracellular AVP peptide was detected by immunocytochemistry. Cultured cells were collected on Vectabond coated slides using a cytospin, fixed in 1% paraformaldehyde and permeabilized with 70% ethanol. Cells were incubated with a primary polyclonal antibody AB1565 to AVP (Chemicon, Harrow, UK) at a dilution of 1 in 1000. This was visualized with a fluorescein isothiocyanate (FITC)-conjugated anti-rabbit IgG (Sigma) and mountant containing DAPI (Sigma) used to counterstain the nuclei.

### Transient transfection of cell lines

A comparison of different transfection methods was undertaken and electroporation selected as an effective method, allowing detection of reporter gene expression in all the established cell lines. Transfection efficiencies were estimated by the flow cytometry method of Fiering et al (1991) to be around 3% in the SCLC and NSCLC cell lines. Single-cell suspensions were prepared either by manual disaggregation (suspension cells) or trypsinization (adherent cells), $7.5 \times 10^6$ cells in a volume of 400 µl RPMI–10% BCS, were used for transfection. An Easiject electroporator (Flowgen), settings: 260 V and 1050 µF (Baum et al, 1994), was used to deliver a total of 40 µg plasmid DNA. This comprised 20 µg of test plasmid (AVP or SV40 promoter (pSV40) coupled to a CAT reporter) and 20 µg of pSVβGal (Promega) to control for transfection efficiency. Cells were transferred immediately to 20 ml of complete media. This method was used for all cell lines with the exception of NHBE, which were transfected using ExGen-500 (TCS Biologicals, Buckingham, UK). NHBE were seeded in 6-well plates at $4 \times 10^5$ cells per well 24 h prior to transfection. Cells were treated with 10 µl ExGen-500 and 2 µg DNA per well in fresh growth media, incubated for 2 h and media replaced.

### Reporter gene assays

Cells were transfected with the reporter constructs as described above, and harvested after a 72-h expression period, in 500 µl of lysis buffer. The protein content of lysates was determined using an ESL protein assay (Boehringer) to confirm yields of cells. CAT and β-galactosidase (βGal) reporter activity were determined using the respective enzyme-linked immunosorbent assay (ELISA) kits (Boehringer) and absorbance converted to pg of enzyme from a standard dilution series. Levels of CAT were calculated as a ratio to pSVβGal reporter expression to standardize for transfection efficiency.

### RESULTS

### Cloning of the AVP promoter and enhancer regions

Although 1.2 kb of the 5′ promoter region for human AVP has been cloned and sequenced (Bahnsen et al, 1992), this has been only partially characterized and no SCLC-specific elements have been identified. We amplified this region by PCR, as described in detail in Methods and shown diagrammatically in Figure 1B. A 1048-bp fragment (–1006 to +42) of the human AVP 5′ region was cloned using *Hin*dIII and *Acc*I sites generated by the primers, this was used to drive expression of a CAT reporter gene. Promoter constructs corresponding to the partially characterized 199-bp

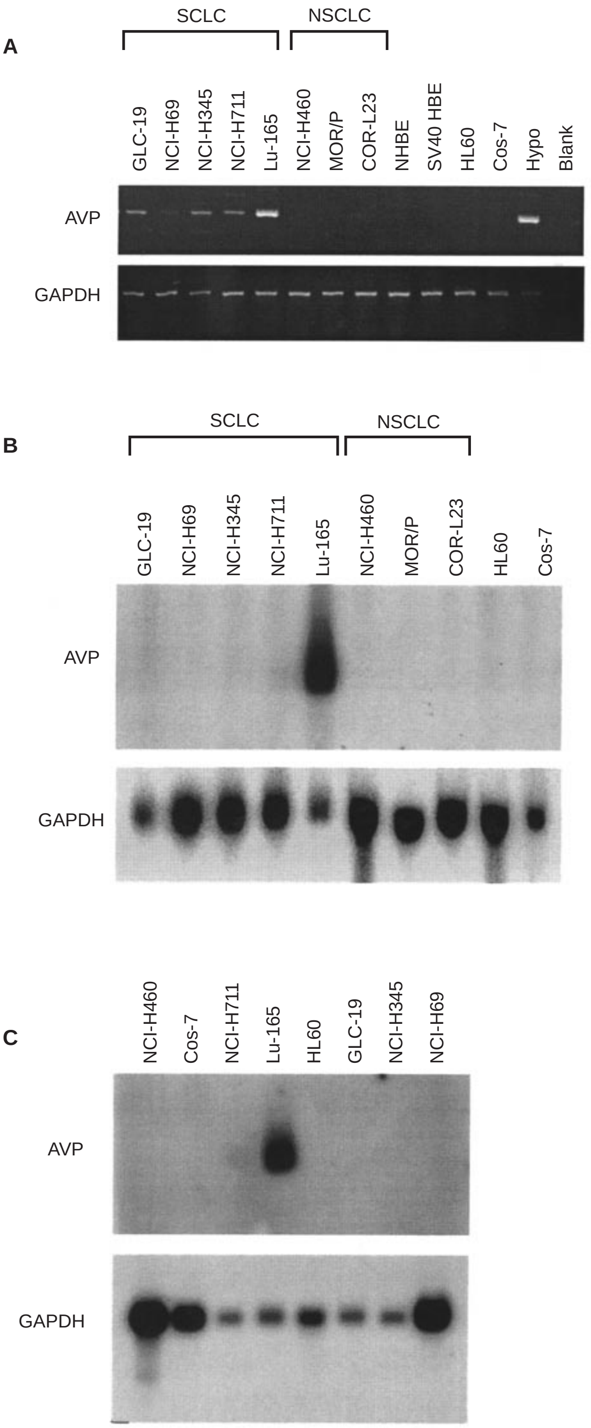

**Figure 2** AVP mRNA expression in cultured cells. AVP expression was determined in human hypothalamus (Hypo), five SCLC cell lines and seven control cell lines: NCI-H460, COR-L23, MOR/P (all NSCLC), NHBE and SV40-HBE (bronchial epithelial cells), HL60 (promyelocytic leukaemia) and Cos-7 (SV40 transformed monkey kidney fibroblasts). (**A**) RT-PCR analysis. Nested PCR with primers specific for AVP (above), GAPDH PCR (below). (**B**) Northern analysis of total RNA, shown hybridized with a probe representing exon 1 of the AVP gene (above), which detected a single transcript of 795-bp. The blot was reprobed for the 1.4-kb GAPDH mRNA (below). (**C**) Northern analysis of poly(A) RNA, shown hybridized with for AVP (above) and GAPDH (below) as described for (**B**)

© 1999 Cancer Research Campaign

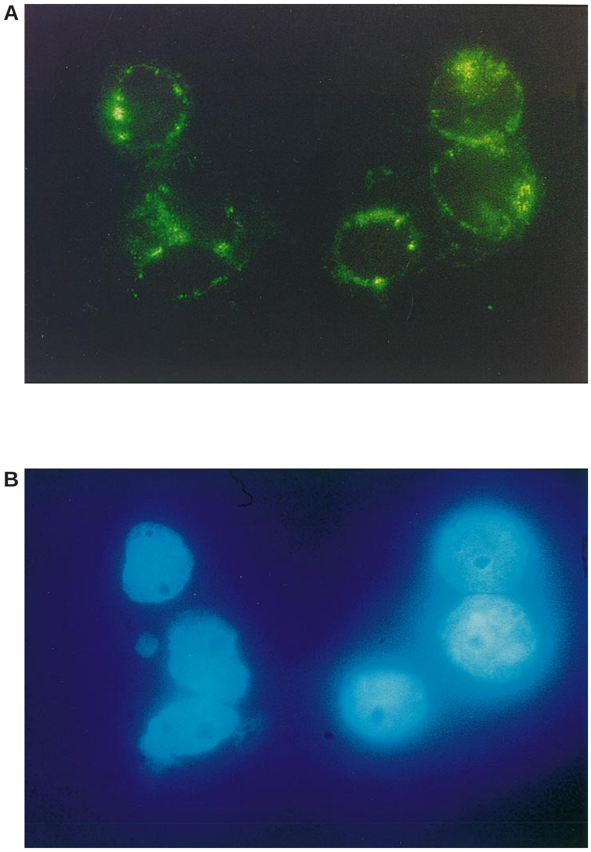

**Figure 3** AVP peptide expression in Lu-165 cells (magnification × 500). (**A**) Immunocytochemistry showing detection of the cytoplasmic peptide. (**B**) Nuclei counterstained with DAPI

region (Figure 1B) and an intermediate 468-bp fragment (Figure 1C), were also cloned for initial experiments.

A region with a high density of transcription factor binding sites, which lacked a TATA box, was identified within the first intron of the human AVP gene by computer analysis. We hypothesized that this region (VEN, Figure 1B) might represent an enhancer element and confer additional tissue-specificity in combination with the 5′ promoter. This was cloned into reporter constructs (as described in Methods) for analysis of enhancer activity in combination with the AVP and tk promoters.

### Detection of AVP expression in lung cancer cell lines

A panel of SCLC and control cell lines was screened for AVP mRNA expression by RT-PCR and Northern analysis (Figure 2). In a 35-cycle standard PCR reaction, an AVP-specific band was only detected in the hypothalamus control and the Lu-165 SCLC line (data not shown). However, by the highly sensitive, but non-quantitative nested RT-PCR method, an AVP-specific PCR product of 227 bp was detected in all the SCLC lines (Figure 2A). This was most intense in the Lu-165 line and identical to that amplified from hypothalamic cDNA. The product was absent in three non-neuroendocrine NSCLC and other control cell lines, including human bronchial epithelial cells. Hybridization of a Northern blot prepared from total RNA, with a probe specific to the first exon of AVP, demonstrated high levels of the AVP transcript in the Lu-165 cell line alone (Figure 2B). An identical result was obtained for a poly($A^+$) mRNA blot prepared from these cells (Figure 2C). These data are in agreement with the results described for the standard RT-PCR protocol. We also examined AVP peptide in the SCLC cell lines by immunocytochemistry. Abundant peptide was seen in the cytoplasm of Lu-165 cells (Figure 3), consistent with earlier reports of peptide secretion (Terasaki et al, 1994), while less intense staining was apparent in the other SCLC lines studied.

Therefore, although AVP mRNA was present in all the SCLC lines tested, the level of mRNA and peptide expression was markedly elevated only in the Lu-165 line. These cells were previously reported to contain 100-fold more AVP peptide than other SCLC lines, such as NCI-H69 by radioimmunoassay (Terasaki et al, 1994). The other SCLC lines expressed low levels of AVP, only detectable by nested PCR, this included NCI-H711, which were originally reported to show high AVP secretion (Gross et al, 1993). However, these findings are consistent with previous observations that high AVP expression, while common in SCLC tumours (Friedmann, 1994), is less common in SCLC lines cultured in vitro (Verbeeck et al, 1992; Gross et al, 1993). It was evident that

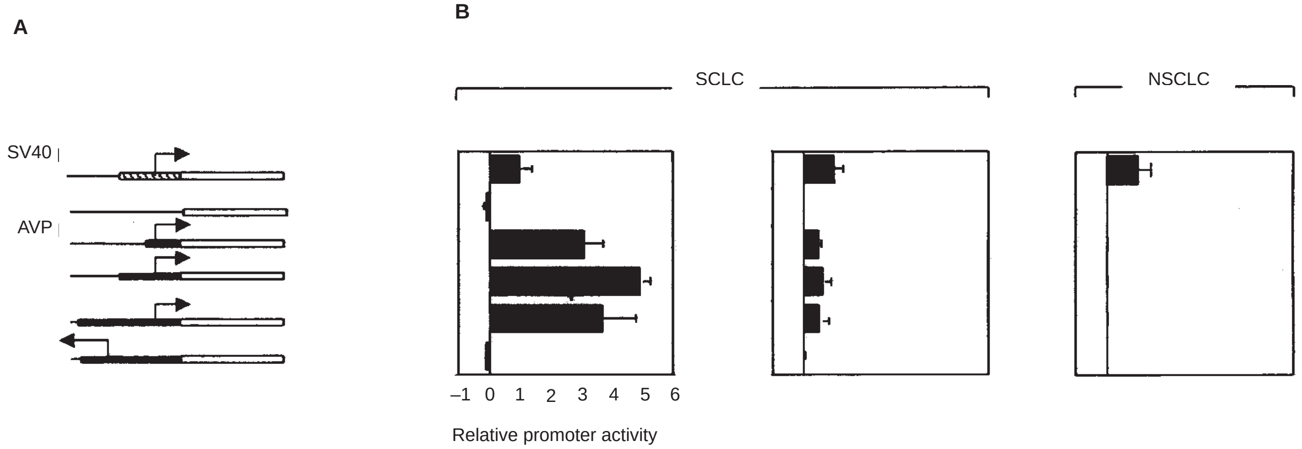

**Figure 4** Relative reporter gene expression in transiently transfected cells. (**A**) The series of three CAT reporter constructs based on the AVP 5′ promoter, SV40 promoter (positive control), reverse orientation AVP promoter and promoterless construct (negative controls). These are illustrated to the left of the corresponding experiments. (**B**) Reporter activity is shown for three representative cell lines: Lu-165 (SCLC, high AVP expression), NCI-H345 (SCLC, low AVP expression) and COR-L23 (NSCLC, no detectable AVP expression). Data are expressed relative to pSV40 (+/– 1 s.d., *n* = 3)

*© 1999 Cancer Research Campaign*

**Table 1** CAT reporter expression in transiently transfected cell lines

| Cell line | Type | AVP RNA | pSV40 | pnone | p199 | p468 | p1048 | p1048 rev |
|---|---|---|---|---|---|---|---|---|
| GLC-19 | SCLC | + | 0.14 (0.14) | –0.26 (0.35) | 0.45 (0.09) | 0.57 (0.08) | 0.35 (0.14) | –0.10 (0.08) |
| NCI-H69 | SCLC | + | 0.60 (0.10) | 0.00 (0.00) | 0.35 (0.11) | 0.24 (0.06) | 0.23 (0.03) | 0.00 (0.01) |
| NCI-H345 | SCLC | + | 0.28 (0.08) | 0.00 (0.01) | 0.15 (0.01) | 0.18 (0.07) | 0.15 (0.05) | 0.00 (0.02) |
| NCI-H711 | SCLC | + | 0.33 (0.08) | –0.01 (0.01) | 0.13 (0.06) | 0.16 (0.07) | 0.09 (0.03) | –0.01 (0.01) |
| Lu-165 | SCLC | +++ | 1.49 (0.60) | –0.18 (0.06) | 4.63 (0.9) | 7.33 (0.5) | 5.51 (1.6) | –0.15 (0.0) |
| NCI-H460 | NSCLC | – | 4.11 (0.94) | 0.00 (0.00) | 0.00 (0.01) | 0.00 (0.00) | –0.01 (0.01) | –0.02 (0.01) |
| MOR/P | NSCLC | – | 0.50 (0.11) | 0.00 (0.00) | 0.00 (0.00) | 0.00 (0.00) | 0.00 (0.00) | 0.00 (0.00) |
| COR-L23 | NSCLC | – | 0.59 (0.24) | 0.00 (0.00) | 0.00 (0.01) | 0.00 (0.01) | 0.00 (0.01) | 0.00 (0.00) |
| NHBE | Lung control | – | 1.41 (1.69) | ND | ND | 0.07 (0.09) | ND | ND |
| HL60 | Suspension control | – | 0.94 (0.39) | 0.03 (0.01) | 0.06 (0.03) | 0.05 (0.03) | 0.05 (0.03) | 0.04 (0.02) |
| Cos-7 | Transfection control | – | 1.08 (0.06) | 0.01 (0.01) | 0.07 (0.04) | 0.12 (0.01) | 0.11 (0.01) | 0.01 (0.01) |

Cells were co-transfected with pSVβGal and either the AVP promoter fragments or an SV40 promoter (pSV40) coupled to the CAT reporter gene. Reporter activity was determined by ELISA as pg ml$^{-1}$ of each enzyme. Levels of CAT were calculated as a ratio to pSVβGal reporter expression to standardize for transfection efficiency, so promoter activity is expressed as CAT:βGal activity. $n$ = 3 for all data (+/– 1 s.d.). ND, not done.

transcription of AVP was markedly SCLC-specific, as no transcript was detected in normal lung epithelium, NSCLC or the other tumour cell lines tested. This panel of cell lines, for which we have characterized endogenous transcription, was used to evaluate reporter gene expression from the cloned AVP promoter.

### Characterization of the AVP promoter

The AVP promoter/CAT reporter plasmids described are represented diagrammatically in Figure 4A. These were co-transfected with pSVβGal to control for transfection efficiency and relative reporter gene expression data are shown for ten cell lines and NHBE in Table 1. All three fragments of the AVP promoter were active in all the SCLC lines. Although the intermediate 468-bp region showed slightly higher activity in the majority of SCLC lines, the large promoter fragments contributed no significant enhancer activity. The promoterless vector and the reverse orientation control had no reporter expression.

In addition, the level of promoter activity in the SCLC cell lines reflected their normal transcriptional activity (Figure 2). High level expression was seen in the Lu-165 cell line, which ranged from 3.1- to 4.9-fold greater than that obtained with the SV40

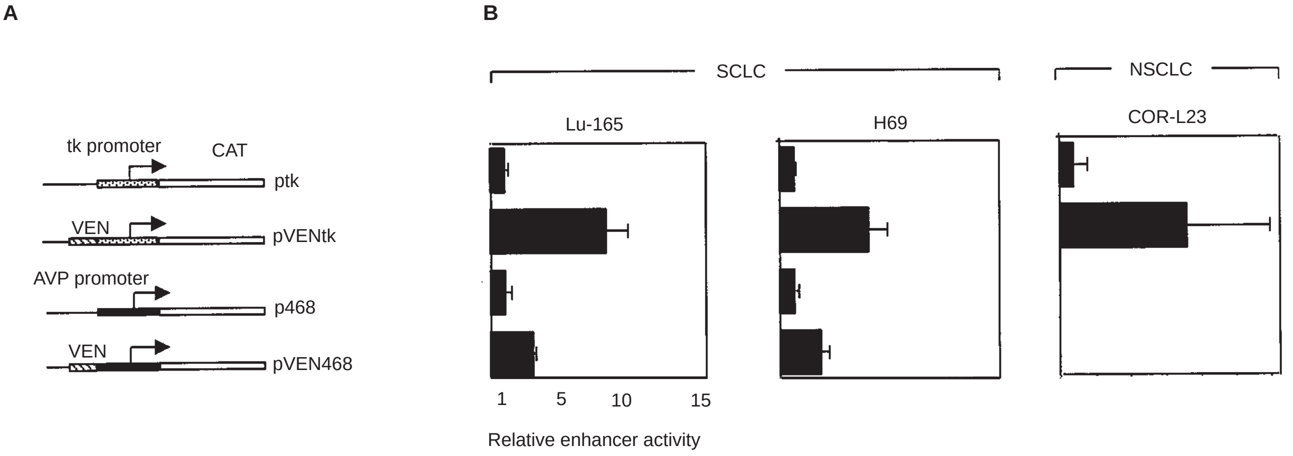

**Figure 5** Relative activity of an enhancer. (**A**) The VEN enhancer region was coupled to the AVP promoter (p468) or a minimal tk general promoter (ptk), these CAT reporter constructs are shown to the left of corresponding experiments. (**B**) Relative enhancer activity in cell lines with varying levels of endogenous AVP expression: Lu-165 (very high), H69 (very low) and COR-L23 (not detected). The mean data for reporter gene expression of each promoter alone (p468 or ptk) is normalized to 1, or represented as 0 if inactive. Enhancer activity is shown relative to the basic promoter activity (+/– 1 s.d., $n$ = 3)

 © 1999 Cancer Research Campaign

**Table 2** Relative enhancer activity in transiently transfected cell lines

| Cell line | Type | AVP | ptk | pVENtk/ptk | p468 | pVEN468/p468 |
|---|---|---|---|---|---|---|
| GLC-19 | SCLC | + | 1 | 2.39 (s.d. = 0.79) | 1 | ND |
| NCI-H69 | SCLC | + | 1 | 6.00 (s.d. = 7.33) | 1 | 2.80 (s.d.=0.56) |
| NCI-H345 | SCLC | + | 1 | 2.94 (s.d. = 0.59) | 1 | ND |
| NCI-H711 | SCLC | + | 1 | 4.68 (s.d. = 0.83) | 1 | 2.54 (s.d.=1.22) |
| Lu-165 | SCLC | +++ | 1 | 7.90 (s.d. = 1.49) | 1 | 2.90 (s.d.=0.21) |
| NCI-H460 | NSCLC | – | 1 | 21.8 (s.d. = 0.86) | 1 | 0.00 (s.d.=0.00) |
| MOR/P | NSCLC | – | 1 | 36.0 (s.d. = 3.00) | 1 | ND |
| COR-L23 | NSCLC | – | 1 | 9.00 (s.d. = 6.00) | 1 | 0.00 (s.d.=0.00) |
| NHBE | Lung control | – | 1 | ND | 1 | ND |
| HL60 | Suspension control | – | 1 | 1.50 (s.d. = 0.14) | 1 | ND |
| Cos-7 | Transfection control | – | 1 | 6.25 (s.d. = 3.50) | 1 | ND |

Activity of the enhancer from intron 1 of the AVP gene in the panel of cell lines. Activity of enhancer constructs (VEN) is shown relative to the same promoter in the absence of the enhancer, either ptk (minimal thymidine kinase general promoter) or p468 (AVP promoter expressed in SCLC only). *n* = 3 +/– 1 s.d. ND, not done.

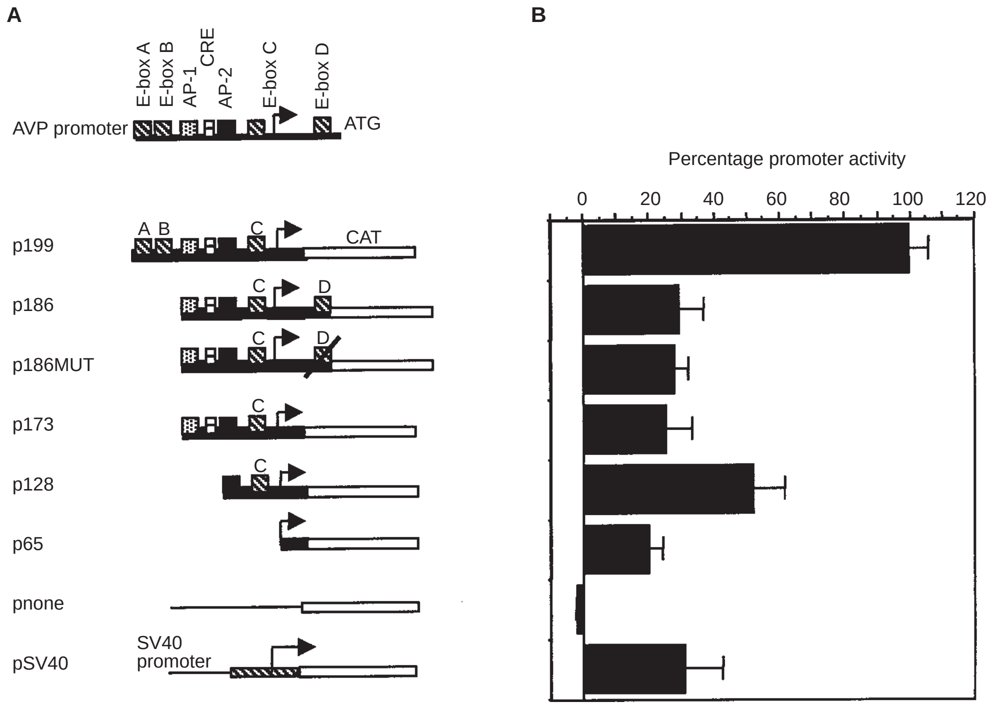

**Figure 6** Investigation of the AVP promoter elements required for restricted expression. (**A**) The proximal AVP promoter showing putative E-box and other transcription factor binding sites (top). The location of these sites, within the parent p199 reporter construct, further 5′ deletions and other modifications of this construct, are shown below. The TATA box is located at –23, potential CRE, AP-1, AP-2 and four E-box sites are illustrated, other potential AP-2 sites coincide with E-box B, the CRE and AP-2 site shown. The constructs p199, p173, p128 and p65 represent –157, –131, –86 and –23 to +42 respectively; p186 and p186MUT (mutation in E box D at +40) represent –131 to +56. (**B**) Histogram showing the relative CAT reporter activity from these constructs following transfection into Lu-165 cells, the activity of p199 is represented as 100% (+/– 1 s.d., *n* = 3)

promoter (Figure 4B). The NCI-H345 SCLC line has lower but clearly detectable AVP mRNA expression (Figure 2A), and the AVP promoter activity was only around half that of the SV40 promoter in these cells. In the NSCLC NCI-H460 cell line (Figure 4B), these AVP promoter fragments were inactive. Indeed in all the AVP-negative lines tested, including NHBE cells, the SV40 promoter was active, whereas the AVP 468-bp promoter had negligible activity (Table 1). Strikingly, even the p199 fragment was able to confer tumour-specificity on gene expression in the panel of cell lines tested.

Additional experiments using a cytomegalovirus (CMV) promoter/βGal reporter to standardize the data yielded similar results. CAT expression for p199 and p1079rev normalized to pSV40 was 2.26 and 0.00 respectively in Lu-165 cells, compared to 0.00 and –0.01 in NCI-H460. Comparable results (of 0.59 and 0.05 for p199 and p1079rev respectively) were also obtained in the NCI-H69 cell line on co-transfecting with the plasmid pRL-CMV and detection of *Renilla* luciferase by the dual-luciferase assay (Promega), indicating the results are independent of the co-transfection plasmid. The transfection protocol had no influence on

© 1999 Cancer Research Campaign reporter gene expression as calcium phosphate precipitation, which efficiently transfected NCI-H69, yielded similar results to those obtained by electroporation in this cell line (data not shown).

### Characterization of a potential enhancer

We confirmed that the VEN region lacked intrinsic promoter activity in the promoterless construct pVEN, where the ratio of CAT to βGal expression ($n$ = 3) was zero in both the Lu-165 and NCI-H460 cell lines. However, when VEN was coupled to other promoters (Figure 5A) it was shown to enhance transcriptional activity of the general tk promoter in all cell lines tested (Table 2). Transcription was increased by two- to eight-fold in the SCLC lines and was elevated to an even greater extent in the NSCLC lines, ranging from nine- to 36-fold; representative examples are shown in Figure 5B. However, when coupled to the AVP promoter, the enhancer was only active in SCLC lines, increasing transcription by two- to three-fold. The AVP promoter remained inactive in the NSCLC lines, such as COR-L23 (Table 2 and Figure 5B), even in combination with this strong enhancer. Therefore, although not itself tumour-specific, the VEN region does not override restricted expression when coupled to the AVP promoter. Interestingly, it substantially increases transcription in SCLC lines, such as NCI-H69 (Figure 5B), where endogenous AVP expression is very low (Figure 2A).

### Further analysis of the 5′ promoter

In contrast to the putative enhancer, the AVP 5′ region only directed strong expression in a SCLC line with high endogenous AVP. We analysed the entire 5′ promoter sequence for transcription factor binding motifs using ‘Signal Scan’. This showed previously reported elements and a number of other potentially important sites. The sites of interest are illustrated in Figure 6A and include AP-1, AP-2 and E-box motifs. The AP-2 sites and cAMP responsive element (CRE) are seen in the homologous region of the rat promoter (Iwasaki et al, 1997). There is a high density of E-box motifs within this region, some of which bind proteins in the rat AVP promoter (Grace et al, 1999).

To elucidate the regulatory elements between –157 and +42, which are involved in the pathological activation of the human AVP promoter in SCLC, we generated additional 5′ deletion constructs, illustrated in Figure 6A. Two E-box motifs (A and B) are located at the 5′ end of the 199 bp fragment, adjacent to an AP-1 site with which they may interact. The p173, p186, p186MUT plasmids all lack E-boxes A and B, while p186 (–131 to +56) includes an additional E-box (D) 3′ to the transcriptional start site. The construct p128 contains only E-box C and p65 represents the minimal promoter.

Reporter gene expression from these plasmids, in transiently transfected Lu-165 cells, is shown in Figure 6B. It is clear that while the loss of E-boxes A and B (p173) results in a reduction of AVP promoter activity to approximately 25% of that for p199, this is still equivalent to the SV40 promoter activity. E-boxes C and D appear to have little influence on promoter activity in this cell line, although in p128, where sequences adjacent to C are removed, the activity increases approximately twofold. This partially restored activity in p128 implies a repressor function within the region –131 to –86. Clearly, some of these elements are involved in modulating expression, and at least some of the E-box motifs increase promoter activity. However, none of these elements are essential for tumour-specificity as none of the deletion constructs described were active in the NSCLC control cell line (data not shown). The minimal p65 promoter, encompassing only the basic transcriptional machinery, retains specificity.

### DISCUSSION

We have demonstrated that short fragments of the human AVP 5′ promoter are sufficient to direct strong, highly SCLC-specific gene expression in vitro. This mimics the activity of the endogenous AVP promoter in a panel of cell lines and the essential regulatory elements are localized between –157 and +42 in the human promoter. Additional sites present between –426 and –157 may account for the marginally increased activity associated with an intermediate fragment, whilst the inclusion of further sequence up to –1006 conveys no additional activation, and may encompass some repressor activity. It was recently reported that elements mediating osmotic and endothelin 3 regulation of the rat AVP promoter may be present between –1500 and –532 (Kim et al, 1996) and a hyperosmolarity responsive element is predicted at –355 in the human AVP promoter (Okazaki et al, 1997). Repressors are likely to play a role in restricting expression, and a glucocorticoid repressor element has been localized to between –300 and –155 in a bovine transgene (Burke et al, 1997). In each case, the homologous regions of the human promoter lie outside the 199-bp promoter construct described here. It therefore is unlikely that any of these elements are involved in SCLC-specific expression, although indirect glucocorticoid repression has been described in the proximal rat AVP promoter (Iwasaki et al, 1997).

Several neuronal transcription factors involved in determination of cell lineage, have been identified as candidate SCLC-specific factors. Those implicated in control of neuropeptide expression belong to two main groups, the POU-domain and the basic helix–loop–helix (bHLH) families. Three E-boxes are common to the cloned fragments initially evaluated and may be involved in binding bHLH factors. Human achaete-scute homologue 1 (hASH-1) is a bHLH factor, which is not expressed in normal adult tissue, but has been shown in neuroendocrine tumours including SCLC (Ball et al, 1993) and as such represents a candidate SCLC-specific factor. Therefore, it was hypothesized that the E-box motifs in the AVP promoter may be bound by such a bHLH SCLC-specific transcription factor, resulting in the AVP expression seen in SCLC.

Interestingly, since this manuscript was submitted, an E-box (CACGTG) in the mouse AVP promoter has been reported to control circadian expression in the suprachiasmatic nuclei (Jin et al, 1999). This corresponds to the human E-box A described here and lends support to our suggestion that it represents a potential tissue-specific enhancer. E-boxes A and B are also adjacent to an AP-1 site, and synergy between AP-1 and E-box sites has been suggested to contribute to tissue-specific regulation (Quinn, 1996; Yoon and Chikaraishi, 1992). Our data indicate that although some E-box motifs are used, additional repressor elements within the minimal promoter (p65) are essential for SCLC-specificity, and a pattern of both positive and negative regulation operates, as described already for other neuropeptide promoters (Quinn, 1996).

We have also described a non-selective intronic enhancer region. The activity of this sequence may be attributable to the predicted presence of Sp1 and AP-2 sites. This region was not SCLC-specific and in fact, was shown to act as a strong enhancer in the NSCLC line. Interestingly though, when the enhancer was coupled to the 468 bp AVP promoter this remained inactive in the

 © 1999 Cancer Research Campaign

NSCLC line, emphasizing the restrictive nature of the AVP 5′ promoter. The ability of the enhancer to increase transcription when coupled to the AVP promoter in a SCLC cell line with low endogenous AVP production (H69) implies, that although this may not naturally be a functional enhancer of the AVP promoter, it represents a useful tool in producing a synthetic promoter construct to target SCLC.

Our findings have potential applications in targeting gene therapy, for example to express suicide genes in a prodrug approach. Other studies have analysed the myc-mad response element (Kumagai et al, 1996) and the CD24 promoter (Pass et al, 1998) as potential SCLC-specific promoters. However, both these approaches have inherent drawbacks and neither is likely to prove as highly SCLC-specific as the AVP promoter. The myc-mad response element is an E-box motif of the specific sequence CACGTG. This is the same sequence as the E-box motif A within the AVP promoter (Figure 6A), which may enhance, but not restrict, expression in our system. In addition Myc is expressed in all cells and CD24 is highly expressed in neutrophils.

Whilst small amounts of AVP may be synthesized elsewhere (Murphy et al, 1993), physiological expression is essentially restricted to the hypothalamus and ectopic production by SCLC in vivo is higher than in vitro. Therefore, anti-tumour treatments targeted at AVP expressing cells would be predicted to have few systemic side-effects. An attraction of the AVP promoter model is that hypothalamic mRNA expression can be reduced by giving a fluid load, protecting the organ from SCLC-directed cytotoxic therapy. Alternatively, replacement therapy is already available, should hypothalamic damage occur. Therefore the AVP promoter is a valuable tool for designing therapies targeted to SCLC.

### ACKNOWLEDGEMENTS

This work was funded by the Association for International Cancer Research (AICR), St Andrews, Scotland and the Cancer Research Campaign (CRC). We thank Ms Jodie Edgson for her excellent technical assistance, and are grateful to Prof. Ian Hart and Dr John Quinn for useful discussions.

### REFERENCES

Ang HL, Carter DA and Murphy D (1993) Neuron-specific expression and physiological regulation of bovine vasopressin transgenes in mice. *EMBO J* **12**: 2397–2409

Bahnsen U, Oosting P, Swaab DF, Nahke P, Richter D and Schmale H (1992) A missense mutation in the vasopressin-neurophysin precursor gene cosegregates with human autosomal dominant neurohypophyseal diabetes insipidus. *EMBO J* **11**: 19–23

Ball DW, Azzoli CG, Baylin SB, Chi D, Dou S, Donis-Keller H, Cumaraswamy A, Borges M and Nelkin BD (1993) Identification of a human achaete-scute homolog highly expressed in neuroendocrine tumours. *Proc Natl Acad Sci USA* **90**: 5648–5652

Baum C, Forster P, Hegewisch-Becker S and Harbers S (1994) An optimised electroporation protocol applicable to a wide range of cell lines. *Biotechniques* **17**: 1058–1062

Burke ZD, Ho MY, Morgan H, Smith M, Murphy D and Carter D (1997) Repression of vasopressin gene expression by glucocorticoids in transgenic mice: evidence of a direct mechanism mediated by proximal 5′ flanking sequence. *Neuroscience* **78**: 1177–1185

Coulson JM, Stanley J, Staff D and Woll PJ (1997) Evaluation of AVP and V1a receptor expression in SCLC cell lines: potential for an autocrine growth loop? *Lung Cancer* **18**: A599

Fay MJ, Friedmann AS, Yu XM and North WG (1994) Vasopressin and vasopressin-receptor immunoreactivity in small-cell lung-carcinoma (SCCL) cell lines – disruption in the activation cascade of V-1a-receptors in variant SCCL. *Cancer Lett* **82**: 167–174

Fiering SN, Roederer M, Nolan GP, Micklem DR, Parks DR and Herzenberg LA (1991) Improved FACS-Gal: flow cytometric analysis and sorting of viable eukaryotic cells expressing reporter gene constructs. *Cytometry* **12**: 291–301

Friedmann AS, Memoli VA and North WG (1993) Vasopressin and oxytocin production by non-neuroendocrine lung carcinomas; an apparent low incidence of gene expression. *Cancer Lett* **75**: 79–85

Friedmann AS, Mallot KA, Memoli VA, Pai SI, Yu X-M and North WG (1994) Products of vasopressin gene expression in small-cell carcinoma of the lung. *Br J Cancer* **69**: 260–263

Grace CO, Fink G and Quinn JP (1999) Characterization of potential regulatory elements within the rat arginine vasopressin proximal promoter. *Neuropeptides* **33**: 81–90

Gross AJ, Steinberg SM, Reilly JG, Bliss DP, Brennan J, Le PT, Simmons A, Phelps R, Mulshine JL, Ihde DC and Johnson BE (1993) Atrial natriuretic factor and arginine vasopressin production in tumour cell lines from patients with lung cancer and their relationship to serum sodium. *Cancer Res* **53**: 67–74

Hara Y, Battey J and Gainer H (1990) Structure of the mouse vasopressin and oxytocin genes. *Mol Brain Res* **8**: 319–324

Huber BE, Richards CA and Krenitsky TA (1991) Retroviral-mediated gene therapy for the treatment of hepatocellular carcinoma: an innovative approach for cancer therapy. *Proc Natl Acad Sci USA* **88**: 8039–8043

Iwasaki I, Oiso Y, Saito H and Majzoub JA (1997) Positive and negative regulation of the rat vasopressin gene promoter. *Endocrinology* **138**: 5266–5274

Jin X, Shearman LP, Weaver DR, Zylka MJ, De Vries GJ and Reppert SM (1999) A molecular mechanism regulating rhythmic output from the suprachiasmatic circadian clock. *Cell* **96**: 57–68

Johnson BE, Chute JP, Rushin J, Williams J, Le T, Venzon D and Richardson GE (1997) A prospective study of patients with lung cancer and hyponatremia of malignancy. *Am J Respir Crit Care Med* **156**: 1669–1678

Kim JK, Summer SN, Wood WM and Schrier RW (1996) Osmotic and non-osmotic regulation of arginine vasopressin (AVP) release, mRNA, and promoter activity in small cell lung cancer (SCLC) cells. *Mol Cell Endocrinol* **123**: 179–186

Kumagai T, Tanio Y, Osaki T, Hosoe S, Tachibana I, Ueno K, Kijima T, Horai T and Kishimoto T (1996) Eradication of myc-overexpressing small cell lung cancer cells transfected with herpes simplex thymidine kinase gene containing myc-max response elements. *Cancer Res* **56**: 354–358

Maier JAM, Voulalas P, Roeder D and Macaig T (1990) Extension of the life-span of human endothelial cells by an interleukin-1α antisense oligomer. *Science* **249**: 1570–1574

Maurer LH, O'Donell JF, Kennedy S, Faulkner CS, Rist K and North WG (1983) Human neurophysins in carcinoma of the lung: relation to histology, disease stage, response rate, survival, and syndrome of inappropriate antidiuretic hormone secretion. *Cancer Treat Rep* **67**: 971–976

Murphy D, Funkhouser J, Ang H-L, Foo NC and Carter D (1993) Extrahypothalamic expression of the vasopressin and oxytocin genes. *Ann NY Acad Sci* **689**: 91–106

North WG, Fay MJ, Longo KA and Du J (1998) Expression of all known vasopressin receptor subtypes by small cell tumors implies a multifaceted role for this neuropeptide. *Cancer Res* **58**: 1866–1871

Okazaki T, Ishikawa T, Nishimori S, Igarashi T, Hata K and Fujta T (1997) Hyperosmolarity-induced gene stimulation is mediated by the negative calcium responsive element. *J Biol Chem* **272**: 32274–32279

Pardy K, Adah RAH, Carter DA, Seah V, Burbach JPH and Murphy D (1992) The identification of a cis-acting element involved in cyclic 3′, 5′ adenosine monophosphate regulation of bovine vasopressin gene expression. *J Biol Chem* **267**: 21748–21752

Pass M, Quintini G, Zarn JA, Zimmermann SM, Sigrist JA and Stahel R (1998). The 5′-flanking region of human CD24 gene has cell-type-specific promoter activity in small-cell lung cancer. *Int J Cancer* **78**: 496–502

Prestridge DS (1995) Predicting pol II promoter sequences using transcription factor binding sites. *J Mol Biol* **249**: 923–932

Prestridge DS (1996) Signal scan 4.0: new databases and sequence formats. *Computer Applications in the Biosciences* **12**: 157–160

Quinn JP (1996) Neuronal-specific gene expression – the interaction of both positive and negative transcriptional regulators. *Progress Neurobiol* **50**: 363–379

Sausville E, Carney D and Battey J (1985) The human vasopressin gene is linked to the oxytocin gene and is selectively expressed in a cultured lung cancer cell line. *J Biol Chem* **260**: 10236–10241

Sethi T and Rozengurt E (1991) Multiple neuropeptides stimulate clonal growth of small cell lung cancer – effects of bradykinin, vasopressin, cholecystokinin, galanin, and neurotensin. *Cancer Res* **51**: 3621–3623

© 1999 Cancer Research Campaign 

Terasaki T, Matsuno Y, Shimosato Y, Yamaguchi K, Ichiniose H, Nagatsu T and Kato K (1994) Establishment of a human small cell lung cancer cell line producing a large amount of anti-diuretic hormone. *Jpn J Cancer Res* **85**: 718–722

Verbeeck MAE, Sutanto W and Burbach JPH (1991) Regulation of vasopressin messenger RNA levels in the small cell lung carcinoma cell line GLC-8: interactions between glucocorticoids and second messengers. *Molecular Endocrinology* **5**: 795–801

Verbeeck MAE, Elands JPM, de Leij LFMH, Buys CHCM, Carney DN, Belper G, Roebroeck AJM, Van de Ven WJM and Burbach JPH (1992) Expression of the vasopressin and gastrin-releasing peptide genes in small cell lung carcinoma cell lines. *Pathobiol* **60**: 136–142

Vile RG and Hart IR (1993) Use of tissue-specific expression of the herpes simplex virus thymidine kinase gene to inhibit growth of established murine melanomas following direct intratumoural injection of DNA. *Cancer Res* **53**: 3860–3864

Woll PJ (1996) Growth factors and lung cancer. In: *Lung Cancer*: *Principles and Practice*, Pass HI, Mitchell JB, Johnson DH and Turrisi AT (eds), pp. 123–131. Lippincott-Raven: Philadelphia

Yoon SO and Chikaraishi DM (1992) Tissue-specific transcription of the rat tyrosine-hydroxylase gene requires synergy between an AP-1 motif and an overlapping E-box containing dyad. *Neuron* **9**: 55–67

*© 1999 Cancer Research Campaign*
